# Intravenous Immunoglobulin Treatment in Chronic Neurological Diseases: Do We Have Maintenance Dose Right?

**DOI:** 10.1155/2014/962530

**Published:** 2014-12-18

**Authors:** Ondrej Dolezal

**Affiliations:** NHS Scotland, Dumfries and Galloway Royal Infirmary, Bankend Road, Dumfries DG14AP, UK

## Abstract

*Objectives*. We tried to define, on individual basis, minimal effective maintenance dose of intravenous immunoglobulins (IVIG) in 26 patients with chronic neurological conditions requiring long-term IVIG treatment. *Methods*. Clinical criteria were reviewed in individual cases (Phase 1) followed by titration phase (Phase 2, 12 months) and posttitration/follow-up phase (Phase 3, 3 months). Objective neurological examination and patient self-reports were used for clinical follow-up. *Results*. 69.2% of patients reported condition as stable, 26.9% as better, and 3.9% as mildly worse. Original mean monthly dose was 1 g/kg; over the period of 12 months we reduced dose of IVIG to mean dose 0.67 g/kg (range 0.3–2.5 g/kg, *P* < 0.0001) which meant reduction by 36.4%. We identified 4 nonresponders and diagnosis in one case was reclassified to degenerative disease. In follow-up phase we reduced dose further to 0.60 g/kg. Cumulative monthly dose dropped from 2040 g to 1298 g and to 991 g, respectively. Financial expenses were reduced significantly (by −36.4% during titration phase and by −51.4% during follow-up phase) (comparing with baseline) (*P* < 0.0001). *Conclusion*. Individual dose titration leads to significant maintenance IVIG dose reduction with preserved clinical efficacy. Maintenance dose below 1 g/kg (in our study around 0.7 g/kg) has acceptable risk/benefit ratio.

## 1. Introduction

Human intravenous immunoglobulins (IVIG) are used in various neurological diseases, especially myasthenia gravis (MG), chronic inflammatory demyelinating polyneuropathy (CIDP), and multifocal motor neuropathy (MMN), and less commonly in paraneoplastic polyneuropathy (PNP), polyneuropathy associated with paraproteinaemia (PAP), and stiff-man syndrome (SMS).

Clinical effects and mechanism of IVIG action remain unclear. In diseases mediated primarily by antibodies, effect of IVIG is based on their neutralising features (forming immunocomplexes, facilitating phagocytosis, etc.). Other effects such as stabilising “information network” within immune system by providing physiological immunoglobulin spectrum, downregulation of endogenous immunoglobulin production, neutralising autoantibodies, opsonization, facilitation of endogenous immunoglobulin catabolism, complement interactions, and T and B cell suppression were mentioned [1–7]. Mechanisms influencing oligodendroglia and remyelination seem to be even more complicated [8–10]; however, these concepts are mostly theoretical and experimental (“in vitro”) with lack of reliable biomarker available for clinical practice. There is currently range of recommendations available about dosing regimens and frequency of maintenance treatment administration (between 0.6 and 2 gm/kg in regular intervals (3–8 weeks, over one or two days)) [11–16]. Most commonly used maintenance dose is 1 g/kg every 3–6 weeks [17, 18]. Some authors were mentioning since 1990s the need of better definition of effective dose based on frequent follow-up and individualised approach [19–21]. There seems to be simple way of how to achieve this: (1) following strict diagnostic criteria (reducing probability of misdiagnosis), (2) regular and frequent clinical follow-up, and (3) readjusting of dose according to clinical progression/development. This approach can lead to lower frequency of side effect, better tolerability/efficacy, and significant financial savings.

## 2. Methods 

**Figure 1:**
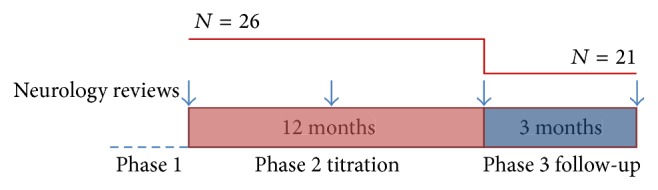
Study design (number of patients, phases).

In our study we observed group of patients (*N* = 26) with various neurological conditions (see Table 1) for 15 months. They were treated by IVIG for more than 3 months at the beginning of observation (range 3–58 months). They all provided oral agreement with IVIG treatment. Patients were on no concomitant immunosuppressive treatment. Aim of our study was to establish minimal effective dose and lowest tolerable infusion frequency without compromising clinical efficacy. At the beginning of our study maintenance dose used in patients was 1 g/kg administered every 4 weeks (as per guidelines of joint task force of the European Federation of Neurological Societies (EFNS) and Peripheral Nerve Society (PNS)) [22–24], and infusion was administered over two consecutive days. That required member of junior medical staff (clerking connected with admission) and there were additional expenses connected with admission. Our study was divided into three separate phases (see Figure 1). In the first phase (Phase 1) general review of currently treated patients was performed; diagnosis and diagnostic criteria (including available test results, lumbar puncture, nerve conduction studies (NCS), etc.) were reviewed as well. All patients were offered to switch to “one-day” infusion (preventing hospital admission). At that point monthly dose never dropped under minimal recommended dose of 0.6 g/kg. This dose allowed us to prevent two-day administration and avoid admission (maximal daily dose administered in one day was 50 g). Intervals were set to 2–6 weeks (on the basis of individual patient experience). Second phase lasted for 12 months and during that period dose and intervals between infusions were changed according to clinical need (titration period). Patients were instructed to report any changes in clinical condition to trained staff and dose was readjusted when needed (month-to-month basis). Infusion room staff (two staff nurses) regularly took part in specialised clinic alongside neurology consultant to provide them with appropriate clinical training. Third phase (lasted 3 months) included another detailed clinical review and further dose adjustments; if clinical condition deteriorated introducing of steroid treatment was considered. Nonresponders were identified. For clinical evaluation subjective information from patients (quality of life, severity of sensory and motor symptoms, etc.) and detailed neurological examination (muscle strength, reflexes, etc.) were used. No serum biomarkers were monitored. Electrophysiology and other diagnostic tests were repeated only if diagnostic doubts were present. For statistical analysis we used Statistica software (StatSoft Inc.) to obtain descriptive statistics and to establish level of significance; *t*-test was used.

## 3. Results

**Figure 2:**
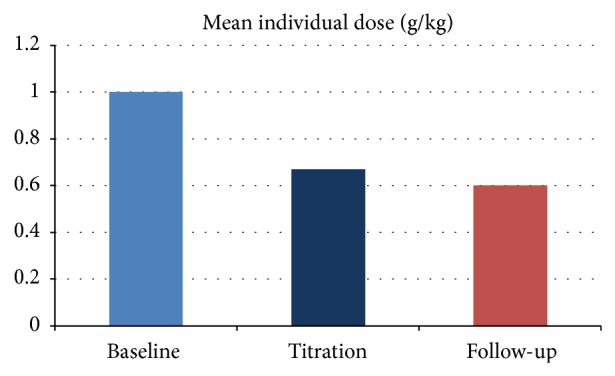
Individual mean dose reduction (g/kg).

All 26 patients agreed on new administration protocol; one patient after two infusions expressed wish to continue with two-day administration (admission). Admission was therefore not needed in 96.1% of patients. If we would use recommended maintenance dose of IVIG (1 g/kg), monthly dose would be 2040 g. Intervals range between infusions was between 2 and 6 weeks and all doses were recalculated to monthly format. Thanks to frequent clinical reviews and appropriate staff training we were able to reduce amount of IVIG to mean dose 0.67 g/kg/month (range 0.3–2.5 g/kg, *P* < 0.0001). So at the end of the second phase (approximately after 12 months) cumulative monthly dose was reduced to 1298 g per month (−36.4% reduction, *P* < 0.000005). Majority of patients were not reporting any significant clinical progression during second phase (69.2% reported their condition as stable, 26.9% as better, and 3.9% as mildly worse) and this agreed with objective examination. 5 patients discontinued treatment at the end of titration period (second phase). Two patients with paraneoplastic polyneuropathy (anti-Hu positive) and one patient with paraprotein mediated polyneuropathy and one with myasthenia gravis discontinued treatment for infectivity (clinical progression or lack of improvement (nonresponders) despite escalation of treatment to maximal dose). One patient was reclassified from multifocal motor neuropathy to degenerative anterior horn cell degeneration/motor neuron disease on the basis of clinical picture and follow-up neurophysiology. Deescalation protocol clearly failed in one patient (CIDP patient) who remained on 2.5 g/kg every month (administered over two days) despite steroids added to treatment regimen. 21 patients entered final observational period and at the end of observation (after further 3 months) mean monthly dose was reduced further to 0.60 g/kg and cumulative monthly dose dropped to 991 gm/month (Figure 2).

Pharmacoeconomic results were surprisingly encouraging as financial expenses were significantly reduced (savings regarding hospital admission are not included in calculation) by 36.4% (*P* < 0.0001) at the end of titration phase. Further decrease to by −51.4% from baseline (*P* < 0.0001) was found at the end of the observation.

From general point of view treatment was well tolerated; during five IVIG administrations some side effects were observed (temporary abdominal discomfort and headaches) (once during the study); in one patient treatment led to chronic eczema reactivation but settled on different IVIG preparation.

### 3.1. CIDP Subgroup

Majority of our patients suffered from CIDP (13 subjects). Their results were copying overall results when mean monthly dose was reduced from 1 g/kg to 0.70 g/kg (*P* = NS) at the end of titration phase and to 0.55 g/kg (*P* < 0.0001) at the end of observation. Clinically patients remained stable or improved (nine and three, resp.) and only one patient deteriorated (deescalation failed; see above).

## 4. Discussion

IVIG are the oldest and most commonly used biological treatment. Currently health services worldwide are under pressure regarding maintaining quality of care and quality of patients' life and simultaneously controlling care costs and expenses. Dosage of IVIG is based on clinical studies mostly from 1990s and EFNS guidelines mentioned possibility of dose reduction (maintenance dose defined between 0.6 and 1 gm/kg per 3–8 weeks and 0.4 and 1.2 g/kg per 2–6 weeks) [17, 25]. Our results proved that investing time and resources to frequent follow-up in patient with chronic autoimmune conditions and adjusting doses (or timely discontinuation in nonresponders) according to clinical outcome are not only leading to significant cost/benefit ratio improvement but also leading to better tolerability of treatment. At least some of the serious side effects (procoagulation and high viscosity state connected with higher risk of cardiovascular, renal events, anaemia, etc.) seem to be dose dependent [26–29]. In our observation we were reducing dose significantly initially however still within recommended guidelines. We duly titrated dose up if needed (range of dose at the end of titrating Phase 2 was 0.3–2.5 g/kg/month). Critics can argue that reduction of IVIG dose was reached by discontinuation of treatment in resistant cases; however all 26 patients were involved in calculation of average dose at the end of titration phase (Phase 2) when mean dose was already reduced to 0.67 g/kg/month. Another criticism could be heterogeneity of the sample. When we analysed subgroup of CIDP patients we found similar findings to other conditions, not statistically different to other diagnoses.

Discontinuation of treatment took place in 5 patients and their condition remained stable in the following three months during Phase 3. Remarkably, but not surprisingly, nonresponders were recruited from the group of paraprotein mediated and paraneoplastic polyneuropathy [30]. Next step could be considering home IVIG treatment as already piloted in some places [31], which would seem to be appropriate for rural and isolated areas. Subcutaneous administration looks as feasible option as well especially in CIDP patients [32, 33].

## 5. Conclusion 

We succeeded in reducing IVIG dose and avoiding hospital admissions in vast majority of our patients. We believe that maintenance dose significantly lower than of 1 gm/kg, administered every 4–6 weeks, could be sufficient when titration process is conducted carefully. Lower dose of IVIG is providing healthy balance between tolerability, admission need, and financial cost.

## Figures and Tables

**Table 1:** Demographics and diagnoses (*N* = 26).

	Number of pts.
CIDP	13
Multifocal motor neuropathy	4
Myasthenia gravis	3
Stiff-man syndrome	1
Paraprotein assoc. neuropathy	1
Paraneoplastic neuropathy	4

Gender (M/F)	11/15
Mean age (yrs)	62.3
Mean weight (kg)	78.5

## Abbreviations

IVIG: Intravenous immunoglobulins
MG: Myasthenia
CIDP: Chronic inflammatory demyelinating polyneuropathy
MMN: Multifocal motor neuropathy
PNP: Paraneoplastic polyneuropathy
PAP: Polyneuropathy associated with paraproteinaemia
SMS: Stiff-man syndrome
NCS: Nerve conduction studies
EFNS: European Federation of Neurological Societies
PNS: Peripheral Nerve Society.
